# A Group-1 Grass Pollen Allergen Influences the Outcome of Pollen Competition in Maize

**DOI:** 10.1371/journal.pone.0000154

**Published:** 2007-01-17

**Authors:** Elene R. Valdivia, Yajun Wu, Lian-Chao Li, Daniel J. Cosgrove, Andrew G. Stephenson

**Affiliations:** 1 Department of Biology and The Plant Physiology Graduate Program, The Pennsylvania State University, University Park, Pennsylvania, United States of America; 2 Proteomics and Mass Spectrometry Core Facility, The Huck Institutes of the Life Sciences, The Pennsylvania State University, University Park, Pennsylvania, United States of America; Massachusetts General Hospital and Harvard Medical School, United States of America

## Abstract

Worldwide, 400 million people suffer from hay fever and seasonal asthma. The major causative agents of these allergies are pollen specific proteins called the group-1 grass pollen allergens. Although details of their antigenicity have been studied for 40 years with an eye towards immunotherapy, their function in the plant has drawn scant attention. Zea m 1 constitutes a class of abundant grass pollen allergens coded for by several genes that loosen the walls of grass cells, including the maize stigma and style. We have examined the impact of a transposon insertion into one of these genes (*EXPB1,* the most abundant isoform of Zea m 1) on the production of Zea m 1 protein, pollen viability, and pollen tube growth, both *in vitro* and *in vivo*. We also examined the effect of the insertional mutation on the competitive ability of the pollen by experimentally varying the sizes of the pollen load deposited onto stigmas using pollen from heterozygous plants and then screening the progeny for the presence of the transposon using PCR. We found that the insertional mutation reduced the levels of Zea m 1 in maize pollen, but had no effect on pollen viability, *in vitro* pollen tube growth or the proportion of progeny sired when small pollen loads are deposited onto stigmas. However, when large pollen loads are deposited onto the stigmas, the transposon mutation is vastly underrepresented in the progeny, indicating that this major pollen allergen has a large effect on pollen tube growth rates *in vivo,* and plays an important role in determining the outcome of the pollen-pollen competition for access to the ovules. We propose that the extraordinary abundance (4% of the extractable protein in maize pollen) of this major pollen allergen is the result of selection for a trait that functions primarily in providing differential access to ovules.

## Introduction

Each silk (stigma/style) of a maize plant can support the germination and growth of numerous pollen tubes, but only one tube enters the micropyle, penetrates the ovule and achieves fertilization. Considering that only the first pollen tube to reach the micropyle passes its genes to the next generation, it is not surprising that the entire process is very fast. In maize, rehydration and germination of the pollen grain occur within 5 min of deposition on the silk, and pollen tubes grow at rates exceeding 1 cm h^−1^ [1]. Even the longest silks that are connected to the lowermost ovaries on an ear are traversed in 24–30 h. To make this trek (up to 40 cm in maize), the male gametophyte must transcribe and translate a large number of genes. In mature maize pollen, an estimated 24,000 genes are expressed by the microgametophyte, of which 10% are pollen-specific [2]. Recent studies of transcript profiling in pollen indicate even higher percentages of pollen-specific gene expression [3], although the vast majority of genes expressed by microgametophytes still appear to be expressed during both the sporophytic and gametophytic stages of the life cycle. Because genes that give a competitive advantage in the race from the stigma to the ovule are expected to increase in the population, it is reasonable to predict that at least some of the pollen-specific genes have evolved in response to pollen-pollen competition for access to the ovules.

The group-1 grass pollen allergens are pollen-specific proteins originally identified by immunologists 40 years ago as the main causative agents of hay fever and seasonal asthma induced by grass pollen [4]–[6]. Although many details of their antigenicity have been studied with an eye towards immunotherapy [7]–[10], their function in the plant has drawn scant attention. Recently these pollen allergens were recognized as members of a subclass of the β-expansin family [11], [12]. Expansins comprise a large superfamily of proteins that characteristically loosen the plant cell wall by weakening the noncovalent bonding of polysaccharides to one another [13]–[16]. The individual members of this superfamily are known to play important roles in plant growth and development [14], [17], [18]. The group-1 allergens from maize pollen, collectively known as Zea m 1, are highly abundant glycoproteins, constituting ∼4% of the protein extracted from pollen; they are rapidly secreted upon pollen hydration and have wall-loosening activity specific for grass cell walls [12], [19]. Because Zea m 1 (and its homologs in other grass species) and the mRNA from the genes that encode it have only been found in grass pollen, it is thought to exhibit pollen-specific gene expression [20], [21]. In this study, we assessed the role of Zea m 1 in pollen viability, pollen tube growth, and pollen competitive ability by use of a maize line containing a *Mu* transposon insertion in *EXPB1* (GenBank Accession AY197353), a gene that codes for Zea m 1d, the most abundantly expressed of four Zea m 1 isoforms [19]. We found that the reduction of Zea m 1 caused by the insertion has a strong effect on pollen tube growth *in vivo* and the ability of pollen to achieve fertilization under conditions of pollen competition.

## Results

### Effects of the *Mu* Insertion on Zea m 1 Production in Pollen

**Figure 1:**
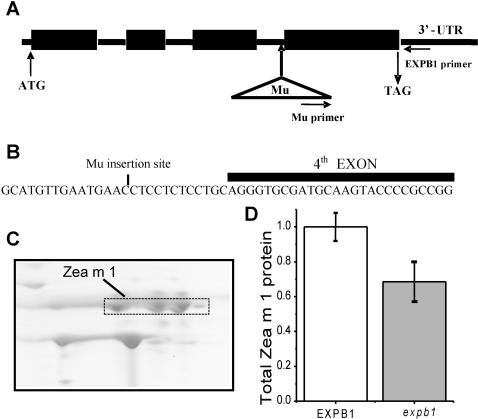
*Mu* insertion into *EXPB1* and its effect on Zea m 1 content of pollen. (a) Cartoon showing the structure of *EXPB1* and location of the *Mu* insertion (exons denoted with boxes). Also indicated are the locations of primers used for PCR screening. (b) *Mu* is inserted near the intron border flanking the fourth exon. (c) Portion of a 2-D gel image of wild type (*EXPB1*) pollen protein showing the Zea m 1 isoforms, which were identified by immunoblotting. (d) Relative amount of total Zea m 1 protein extracted from pollen of *EXPB1/EXPB1* and *expb1/expb1* plants. (Mean±SE; N = 2; t = 9.15; p = 0.035).

From a large library of maize lines bearing Robertson's Mutator (*Mu*) insertions (obtained from Pioneer Hi-Bred International, Inc., Johnson, IA, USA; see [22]–[24]), we identified a single line with a *Mu* insertion in *EXPB1* (Figure 1). From this line we created, via repeated backcrosses into the non-mutator parental line and then self pollinations, true breeding mutant plants (*expb1/expb1*) and wild type plants (*EXPB1/EXPB1*), as well as heterozygous (*EXPB1/expb1*) plants by crossing the true breeding plants. Analysis of pollen protein extracts by two-dimensional gel electrophoresis and immunoblotting from *EXPB1/EXPB1* and *expb1/expb1* plants revealed that overall Zea m 1 production was reduced by 31% in *expb1* pollen compared with the overall production of Zea m 1 in *EXPB1* pollen (Figure 1c,d). It should be noted that we did not expect Zea m 1 production or even the Zea m 1d isoform, to be completely eliminated in the pollen from *expb1/expb1* plants because multiple genes contribute to the Zea m 1 pool and recent evidence indicates that additional genes highly similar to *EXPB1* (>98.8% nucleotide sequence identity) also code for the same isoform [19], [21].

### Effects of the *Mu* Insertion on Pollen Viability and Pollen Performance

**Figure 2:**
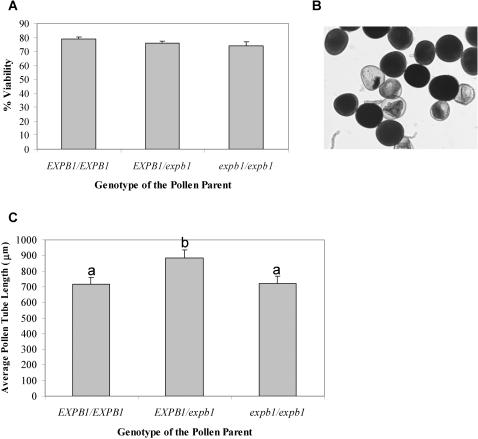
Pollen viability and pollen performance *in vitro* and *in vivo*. (a) Percentage of viable pollen, based on staining with thiazolyl blue (mean±SE, N = 20–22 plants). (b) Micrograph of pollen stained with thiazolyl blue. Viable pollen stained dark purple. (c) Pollen tube growth *in vitro* (mean±SE, N = 20–22). Bars with different letters of the alphabet differ significantly using Tukey pairwise comparisons with the overall probability adjusted for multiple comparisons.

Thiazolyl blue staining of pollen revealed that the reduction in the overall pool of Zea m 1 in *expb1* pollen does not seem to affect the viability of the pollen produced by *expb1/expb1* plants. An analysis of variance (ANOVA) revealed no significant effect of plant genotype on pollen viability (F_2,59_ = 0.92; p = 0.4). For each of the three maize genotypes (*EXPB1/EXPB1, EXPB1/expb1, expb1/expb1*) 75–78% of the pollen stained a deep purple (Figure 2a,b).

When pollen from 20–22 plants from each of the 3 genotypes were germinated and grown on a medium in Petri plates [25], there were significant effects of plant genotype on the mean per plate *in vitro* growth of pollen (ANOVA, F_2,59_ = 4.97; p = 0.01). Tukey pairwise comparisons with adjusted probabilities for multiple comparisons revealed that there was no significant difference in the *in vitro* growth of pollen tubes from *expb1/expb1* and *EXPB1/EXPB1* plants but that the pollen from the *EXPB1/expb1* plants grew faster *in vitro* (Figure 2c) most likely a general result of heterosis, reflecting the hybrid's greater vigor and ability to provision the pollen grains during development (see [26]–[28] for a discussion of the effects of nutrient and energy storage compounds provided by the paternal sporophyte during development on initial pollen tube growth rates and for examples of other species which exhibit similar (i.e., heterosis/inbreeding) effects on pollen tube growth). The lack of a significant difference between the two true breeding lines, however, indicates that *in vitro* pollen tube growth is not affected by the *Mu* insertion. Moreover, there is no hint of a bimodal distribution in the *in vitro* growth of the pollen tubes from the heterozygous plants as would be expected if *EXPB1* and *expb1* pollen grew at different rates *in vitro* (data not shown).

**Figure 3:**
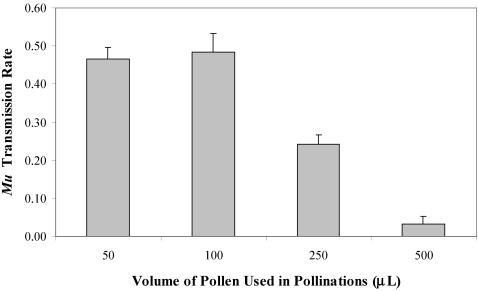
Transmission rate of *expb1* as a function of the size of the pollen load from *EXPB1/expb1* plants (mean±SE, N = 4).

To determine the ability of *EXPB1* and *expb1* pollen to achieve fertilization under conditions of pollen competition, we varied the volume (number) of pollen grains from *EXPB1/expb1* plants deposited onto the silks of *EXPB1/EXPB1* plants. We found that the transmission of *expb1* depended upon the volume of pollen grains used in the pollination (χ^2^ = 53.2; df = 3; p<0.0001) (Figure 3). When the 50 and 100 µL volumes were used in the pollination, fertilization was random (nearly 1∶1) with respect to the *EXPB1* allele carried by the pollen. In contrast, when the two largest volumes of pollen were used in the pollination, the *expb1* gene was significantly underrepresented in the progeny (only 3% of the seeds following pollination with the largest volume), indicating that as the intensity of pollen competition increases, the proportion of seeds sired by *expb1*-bearing pollen decreases.

**Figure 4:**
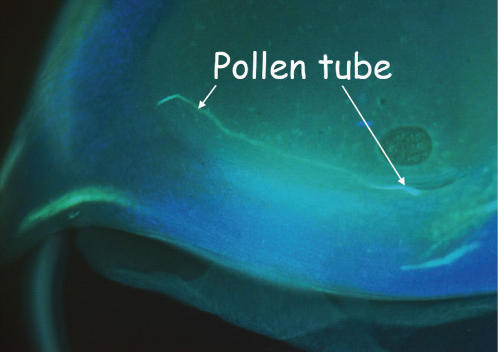
Pollen tube from *EXPB1* pollen growing through ovary tissue for 22 h after pollination. Ovaries were stained with 0.1% aniline blue for 30 min and then examined under a fluorescence microscope.

Experiments designed to directly examine *in vivo* pollen tube growth rates indicate that these differences in the ability to achieve fertilization under competitive conditions are due to differences in pollen tube growth rates. When we examined the silks at 8 h after pollination, we found that silks pollinated with *EXPB1* pollen contained a significantly greater number of pollen tubes (2.18±0.09; mean±SE) at 8 cm below the site of pollen deposition than silks pollinated with *expb1* pollen (0.98±0.06) (ANOVA, F_1,7_ = 120.9; p<0.0001). At 22 h after pollination, we found pollen tubes in 37.5% of the ovaries following pollinations by *EXPB1* pollen (Figure 4). In contrast, we found no pollen tubes in the ovaries at 22 h after pollination when the silks were pollinated by *expb1* pollen. Together these data indicate that the *expb1* pollen grows more slowly *in vivo* than the *EXPB1* pollen.

## Discussion

Competition among males for access the ova/ovules of females is thought to have shaped the haploid phase of the life cycle in both animals (the ejaculate and sperm) and higher plants (the male gametophyte/pollen) (see [29] for recent review). In plants, the pollen load that accumulates on a stigma frequently consists of the pollen from several individuals and often exceeds the number of grains necessary to fertilize all of the ovules [XPATH ERROR: unknown variable "start".]–. Consequently, the pollen from various individuals is placed into a competitive circumstance of great evolutionary importance: only those pollen grains that germinate and grow the fastest through the maternal tissue of the stigma, style and ovary will penetrate the ovule and fertilize the egg. Our data indicate that *EXPB1* plays a large role in generating the rapid *in vivo* growth rates of maize pollen tubes.

Our findings reveal that a 31% reduction in Zea m 1 caused by a transposon insertion into the *EXPB1* gene (the most abundant isoform of Zea m 1) has no significant effects on pollen viability or *in vitro* pollen tube growth rates but has a large effect on *in vivo* pollen tube growth rates and the ability to achieve fertilization under conditions of pollen competition. Because the pollen competition experiment varied the size of the pollen load produced by heterozygous (*EXPB1/expb1*) plants rather than using the pollen produced by the true breeding (homozygous) plants, the results are not an artifact of differences in vigor between the true breeding lines. This experimental design, however, does not exclude the possibility that loci distinct from *EXPB1* could account for our results. After backcrossing the original line with the *Mu* insertion to the non-mutator parental line for 3 generations, the resulting plants still contain, on average, 12.5% of the genes from the original *Mu* insertion line. The vast majority of these genes would randomly segregate into the *EXPB1* and *expb1* pollen produced by the heterozygous plants used in the pollen competition experiment. Only those genes that consistently cosegregate with *expb1* could potentially influence performance of *expb1* pollen. Because only 3% of the seeds produced by our largest pollen load contained *expb1*, another mutation that is responsible for the large effect on pollen performance that we observed would have to lie no more than 3 cM away from the *EXPB1* locus to cause this skewed ratio. The *EXPB1* locus is located on chromosome 9 which has a genetic distance of approx. 150 cM [35]. Consequently the region that cosegregates with the *EXPB1* locus represents <4% of the genetic distance of chromosome 9 in maize (chromosome n = 10). It would be extremely unlikely to find in this small region a second mutation with the precise phenotype described in this paper.

This caveat aside, our findings suggest that the β-expansin encoded by *EXPB1* does not perform a vital role in pollen development or in the internal growth processes of the pollen tube per se. Previous *in vitro* studies demonstrated that Zea m 1 loosens the cell walls of silks and other studies showed that Zea m 1 is secreted by pollen upon hydration and tube growth [12], [19]. These findings support the inference that Zea m 1 assists pollen tube penetration by loosening the maternal cell walls of the stigma/style. Our data also provide strong, additional evidence that *EXPB1* is gametophytically expressed because the performance of pollen from heterozygous plants depended upon whether the pollen carried the *EXPB1* or the *expb1* allele. If the protein (or the mRNA) from *EXPB1* was sporophytically produced (e.g., by the tapetum) and then moved into the pollen, there would be no difference in the performance of pollen bearing different alleles. A few pollen expressed genes have already been shown to affect pollen tube growth rates *in vivo* (see reviews by [36], [37]) including genes that are expressed during both stages of the life cycle (e.g., [38], [39]) and a few pollen specific genes that play important roles in the internal growth processes of the pollen tube [40], [41]. In addition, several studies have shown for genes expressed during both stages of the life cycle that selection on the microgametophyte (e.g., for cold tolerance or herbicide resistance) can alter the proportion of progeny with the selected trait (e.g., [32], [42]–[46]).

Although expansins comprise a large family of genes whose proteins play diverse roles in plant growth and development [14], [17], [18] only in grass pollen have expansins been found to accumulate in such abundance. Given that thousands of genes are expressed in pollen, it is surprising that one protein would constitute 4% of the extracted protein. Because amino acid and protein assembly are expensive [47] in terms of both energy and nitrogen (a nutrient that frequently limits growth and reproduction in plants), it is reasonable to assume that such a large allocation to one type of protein would involve tradeoffs with other aspects of growth, survival and/or reproduction in grasses.

Group-1 pollen allergens (β-expansins) have been detected in every grass [*Poaceae*] species in which they have been examined (mostly turf, pasture, and agricultural grasses, e.g., [48]), but little is known about the quantitative variation within and among species. Our study indicates that the *in vivo* performance and competitive ability of maize pollen varies with the amount of Zea m 1, and we propose that the copious production of this allergenic class of expansin in grass pollen is the evolutionary result of pollen-pollen competition. That is, the pollen tubes that have the most Zea m 1 have first (and thus greater) access to the stylar resources necessary for their growth [28], [37] and, ultimately, for their ability to gain access to ovules. Consequently, there would be strong selection on production of this β-expansin. Tests of this conjecture should include studies of pollen performance using natural variation in the quantity of group-1 pollen allergens within wild populations of grass species, comparative studies to detect selection on the sequences of the genes that contribute to the Zea m 1 pool in maize, and comparative studies to detect selection on the sequences of orthologous group-1 pollen allergen loci among grass species.

Because of the association that we observed between Zea m 1 production and breeding success under conditions of pollen competition, it would be difficult for plant breeders to develop and maintain low allergenic cultivars of turf, pasture, and agricultural grasses. This does not bode well for the 400 million people worldwide who suffer from hay fever and seasonal asthma due to this abundantly expressed β-expansin (the major group-1 grass pollen allergens) [49], [50].

## Material and Methods

### Generation of True Breeding Lines

Maize plants with the *Mu* insertion in *EXPB1* (obtained from Pioneer Hi-Bred International, Inc., Johnson, IA, USA; see [22]–[24]) were backcrossed to the non-mutator inbred parental line (FR696 from Pioneer Hi-Bred International, Inc.) for 3 generations. The presence of *expb1* allele in plants was tracked using a PCR method. Leaf DNA was extracted using a rapid prep protocol [51]. DNA from individual plants was analyzed by PCR. Three primers (*Mu* primer = 5′AGAGAAGCCAACGCCAWCGCCTCYATTTCGTC 3′, EXPB1-5′ primer = 5′AGAATTGGACGTTGGAAGTGTAGAC 3′, EXPB1-3′ primer = 5′CACTCTTTGGAATTCGATCATGAA3′; Fig. 1a,b) were used to discriminate between plants with the *Mu* insertion and plants without it. To identify homozygous *expb1/expb1* lines, we analyzed the segregation of the *EXPB1-Mu* insertion (*expb1* allele) in the progeny of plants that were both self pollinated and crossed to wild type (*EXPB1/EXPB1*) silks. Ears were allowed to set seed and progeny of both crosses were screened for the presence of *Mu* in *EXPB1* (see [21]).

### Zea m 1 Extraction and Analysis

In order to assess the impact of the *Mu* insertion into the *EXPB1* gene on Zea m 1 production, maize pollen was collected in July 2004 from *EXPB1/EXPB1* and *expb1/expb1* plants grown at The Pennsylvania State University Agricultural Experiment Station at Rock Springs PA (near State College, PA), cleaned by passing through a series of sieves, and stored separately at −80°C. Approximately 25 mg of maize pollen was extracted in 4 volumes (0.1 mL) of 50 mM sodium acetate, pH 4.5, for 1 h at 4°C. The extract was centrifuged at 20,800 g for 10 min. Proteins in the supernatant were quantified colorimetrically with the Coomassie Plus® Protein Assay Reagent (Pierce, Rockford, IL) according to the manufacturer's instructions.

These proteins were then subjected to 2-dimensional gel electrophoresis. For the first dimension – isoelectric focusing (IEF) – Immobiline DryStrip gels (pH 6–11, 11 cm) and IPG buffer (pH 6–11) were obtained from GE Healthcare Bio-Sciences Corp. (Piscataway, NJ). The gels were rehydrated for 16 h with the rehydration buffer (8 M urea, 2% CHAPS, 0.5% IPG buffer, 0.002% bromophenol blue) containing the protein extracts and then were focused in a PROTEAN IEF cell apparatus (Bio-Rad Laboratories, Hercules, CA) at the following program: running temperature: 20°C; maximum current: 50 µA/gel; Step 1: 200 V for 30 min (linear ramp); Step 2: 300 V for 30 min (rapid ramp); Step 3: 8,000 V for 150 min (linear ramp); Step 4: 8,000 V for 55,000 Vh (linear ramp). After the completion of IEF, the gels were incubated for 15 min in SDS equilibration buffer (50 mM Tris-HCl, pH 8.8, 6 M urea, 30% glycerol, 2% SDS, and 0.002% bromophenol blue) with 10 mg/mL dithiothreitol and then switched into the same buffer containing 25 mg/mL iodoacetamide for another 15 min. For the second dimension, proteins were separated by discontinuous SDS-PAGE in a Criterion Dodeca Cell apparatus (Bio-Rad Laboratories, Hercules, CA) using 12.5% precast gels. 2-D gels were stained for protein with SYPRO Ruby (Bio-Rad Laboratories, Hercules, CA) according to the manufacturer's instructions and quantified using a laser scanner (Molecular Imager FX Pro PLUS from Bio-Rad) and 2-D image analysis software (PDQuest Version 7.3 from Bio-Rad). The protein marker, Mark12 Unstained Standard, for SDS-PAGE, was from Invitrogen Inc. (Carlsbad, CA; Catalog No. LC5677).

To identify the β-expansins, the resulting 2-D gels were then subjected to immunoblot analysis. This analysis was performed in a Bio-Rad Criterion blotter as described by Li et al. [19]. For immunodetection of Zea m 1, both monoclonal and polyclonal antibodies against Lol p 1 were used. Lol p 1 is the group-1 allergen of perennial ryegrass pollen and has a sequence similarity to Zea m 1. The SeeBlue Plus2 Pre-Stained Standard was purchased from Invitrogen (Carlsbad, CA. Catalog No. LC5925).

### Pollen Viability, and Pollen Tube Growth *In Vitro* and *In Vivo*

To assess the impact of the *Mu* insertion into *EXPB1* on pollen viability, we collected pollen at anthesis from 20–22 plants from each of the three genotypes (*EXPB1/EXPB1* (N = 20), *EXPB1/expb1* (N = 22), *expb1/expb1* (N = 20) plants), stained it with thiazolyl blue to assess membrane integrity—a trait that is highly correlated to germinability [52], and counted the number of stained pollen grains in a sample of 100 grains per plant. Pollen of the appropriate genotype was placed onto a slide, stained under a cover slip and observed under a microscope at 50×. The first 100 grains were scored in a left to right transect starting in the left center of the cover slip. We also assessed the *in vitro* growth of pollen tubes from each of the three genotypes by sprinkling the pollen from 20–22 plants of each genotype onto Petri plates containing a maize pollen germination and pollen tube growth media [25]. After 20 minutes at 28°C a few drops of 80% ethanol was added to each plate to stop pollen tube growth; the Petri plates were placed under a dissecting scope at 8×; and the lengths of the first 20 pollen tubes encountered in a left to right transect starting in the left center of the plate were recorded using image analysis [53].

To determine the effect of the *Mu* insertion into *EXPB1* on the competitive ability of pollen *in vivo*, we experimentally manipulated the intensity of competition between mutant and wild type pollen. Pollen from field grown, heterozygous (*EXPB1/expb1*) plants was collected, cleaned, aliquoted into 50, 100, 250 and 500 µL volumes, and sprinkled over virgin silks of true breeding wild type plants (four replicate pollinations per volume, yielding 16 ears). We reasoned that under conditions of intense pollen competition (e.g., the 500 µL sample of pollen) only the fastest growing pollen tubes would achieve fertilization, whereas under conditions of little or no pollen competition (e.g., the 50 µL sample) both the fast and slowly growing pollen would achieve fertilization. A random sample of 30 seeds from each of the 16 ears (480 progeny total) was assessed for the presence of the mutant allele (*expb1*) by PCR.

We also directly examined the growth of *EXPB1* and *expb1* pollen tubes through maize silks. We pollinated the silks of 16 wild type plants (*EXPB1/EXPB1*) with pollen from either *EXPB1/EXPB1* or *expb1/expb1* plants. On eight plants (four from each type of pollination), we removed the silks after 8 h; stained the silks with 0.1% aniline blue for 30 min and examined 10–14 silks from the central region of each ear for the presence of pollen tubes within the region from 7.5 to 8.5 cm from the site of pollen deposition using fluorescence microscopy [54]. On the remaining eight plants we examined 10 ovaries from the central region of the ear for the presence of a pollen tube at 22 h after pollination by excising the ovary, staining it with aniline blue, and examining it under a fluorescence microscope [54].
